# Magnetic resonance imaging and contrast-enhanced ultrasound findings of a recurrent primary breast angiosarcoma: A case report

**DOI:** 10.1097/MD.0000000000024625

**Published:** 2021-02-05

**Authors:** Ou Ou Yang, Tian Lan, Jun Ling He, Hai Bin Xu, Liang Hao, Chang Shu, Zu Jian Hu, Hua Luo

**Affiliations:** a Department of Breast Surgery; b Department of Radiology; c Department of Pathology, Hangzhou Hospital of Traditional Chinese Medicine, Hangzhou, Zhejiang Province, China.

**Keywords:** angiosarcoma, breast, contrast media, magnetic resonance imaging, ultrasonography

## Abstract

**Rationale:**

Primary breast angiosarcoma (PBA) is a rare and overly aggressive entity and account for less than 1% of all breast cancer cases. PBA had a high rate of delayed preoperative diagnosis due to absent distinctive radiographic characteristics.

**Patient concerns:**

We report a case of a 47-year-old female patient who had a previous history of luminal cancer in the right breast with mastectomy; the patient complained of asymmetrically diffuse enlarged, accompanying with a painless mass in the left breast 12 years after the mastectomy of her right breast.

**Diagnoses:**

The tumor mimicked idiopathic granulomatous mastitis on magnetic resonance imaging (MRI) at the first presentation. Contrast-enhanced ultrasound (CEUS) was performed for further lesion characterization and showed heterogeneous rapid hyper enhanced. An ultrasound-guided core needle biopsy was performed, and the pathology report indicated a breast angiosarcoma.

**Interventions:**

The patient underwent a nipple-sparing simple mastectomy with immediate reconstruction of the left breast.

**Outcomes:**

After 8 months later, the tumor recurred, CEUS and MRI examination suggested PBA recurrence, then re-excision with implant removal was performed, the patient had a lung metastasis 4 months later eventually died 22 months after diagnosis.

**Lessons:**

It is not easy to diagnose PBA with the radiographic examination. This case's importance is by combining CEUS and MRI to reflect enhanced morphology and hemodynamic characteristics of PBA and help diagnose breast angiosarcomas.

## 1 Introduction

Breast angiosarcoma is an overly aggressive entity originating from an endothelial cell of the blood vessel wall, rare, and accounts for less than 1% of all breast malignancy.^[1]^ Primary breast angiosarcoma (PBA) is extremely rare, and there are no distinctive radiological features of angiosarcomas on mammogram, sonography, or magnetic resonance imaging (MRI),^[2–5]^ which leading to the preoperative diagnosis is often difficult. Here, we report a case of recurrent PBA in a 47-year-old female patient with detailed pathologic and radiologic records by combining a contrast-enhanced ultrasound (CEUS) and MRI examination and review the literature to describe its imaging features to minimize delayed diagnosis.

## 2 Case report

A 47-year-old Chinese premenopausal woman developed a breast lump at the left breast's retro-areolar and medial region for 10 months. In the last 2 months before the current hospitalization, the tumor rapidly grew and appeared with the bruising of the left breast's overlying skin. She had been previously treated for luminal cancer in the right breast with mastectomy at 35 years of age, and immediate reconstruction of the right breast was performed during the procedure, placing a retro pectoral cohesive gel implant, then followed by adjuvant hormonotherapy with tamoxifen for 5 years. There was no history of breast irradiation. The patient did not suffer from chronic lymphedema. She had no family history of breast or ovarian cancer.

**Figure 1:**
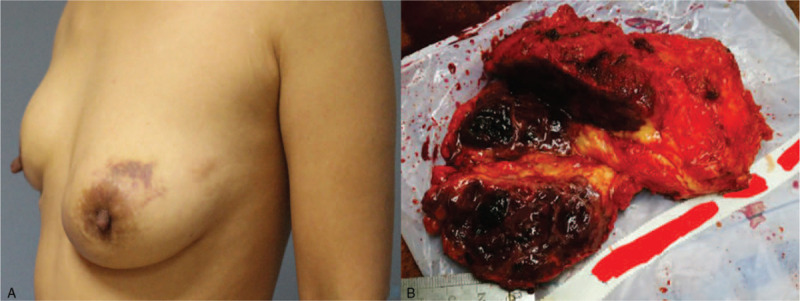
The appearance of the left breast with primary breast angiosarcoma in a 47-year-old female. (A) Bruising of the overlying skin of the left breast. (B) On the mastectomy specimen section of the left breast, the tumor was ill-defined, soft, and fragile, measuring, with a focal area of hemorrhage.

On physical examination, the left breast was asymmetrically diffuse enlarged, without nipple retraction. The patient had a hard, painless mass with limited mobility in the central and medial region of the left breast, along with apparent bruising discoloration of the overlying skin (Fig. 1A), 10.0 × 8.0 × 8.0 cm in diameter. No enlargement of axillary lymph nodes and no skin thickening were found. The right breast was unremarkable.

**Figure 2:**
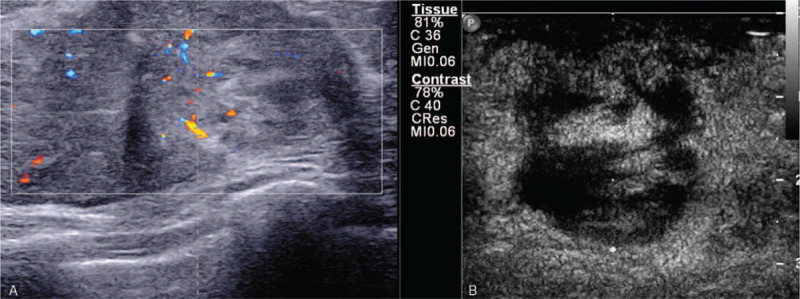
(A) B-mode and color Doppler ultrasound images of the left breast showed a diffuse inhomogeneous isoechoic, well-defined lesion and blood vessels within the lesion. (B) Contrast-enhanced ultrasound images showed the mass an extensive area of perfusion defects.

The B-mode and color Doppler ultrasound showed an inhomogeneous isoechoic and well-defined lesion (Fig. 2A) measuring 9.8 × 7.8 × 3.2 cm at the retro-areolar and medial region of the left breast, which showed no posterior acoustic shadowing; it was reported as Breast Imaging Reporting and Data System (BI-RADS) 4A. CEUS was performed for further lesion characterization. On CUES images, the mass displayed diffuse heterogeneous rapid hyper enhanced, with washout gradually, and the expansion of the enhancement range detected by CUES range is more significant than the 2-dimensional ultrasound. An extensive area of perfusion defects (Fig. 2B) and penetrating surrounding vessels was noted. It was upgraded to BI-RADS 4C.

**Figure 3:**
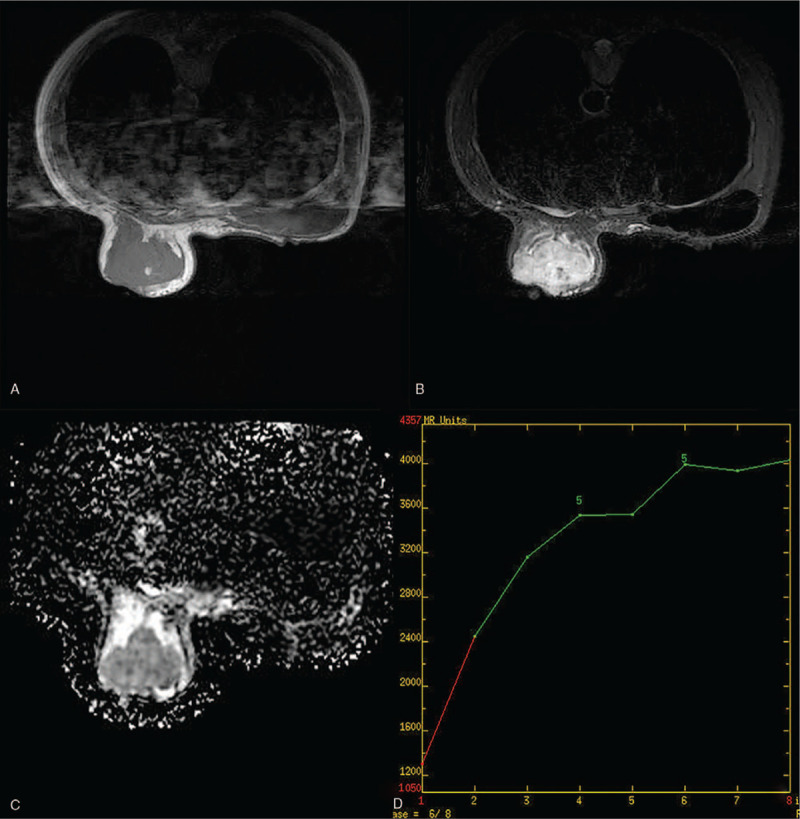
Breast magnetic resonance imaging of a 47-year-old woman with primary breast angiosarcoma of the left breast. (A) Hypointense with scattered mixed high signal intensity foci on T1-weighted images. (B) Hyperintense on T2-weighted images. (C) High apparent diffusion coefficient value. (D) Dynamic contrast-enhanced imaging with time signal intensity curves for type 1.

**Figure 4:**
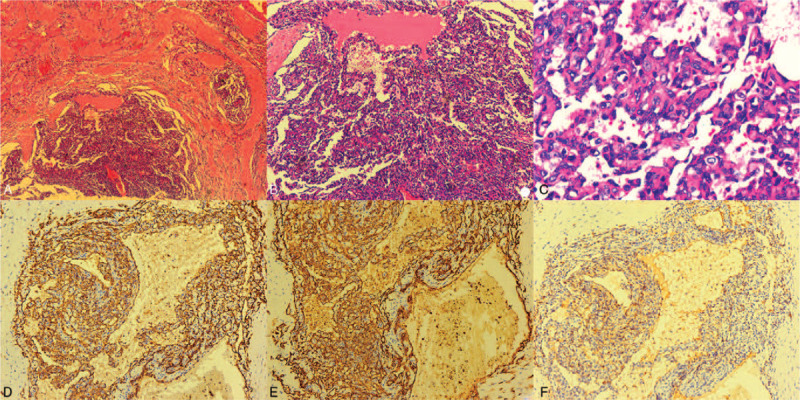
Microscopic examination of the left breast primary breast angiosarcoma (hematoxylin and eosin stain). (A) Magnification power, ×40; (B) Magnification power, ×100; (C) Magnification power, ×400. Immunohistochemistry of the left breast primary breast angiosarcoma. (D) CD31, magnification power, ×100. (E) CD34, magnification power, ×200. (F) Factor VIII, magnification power, ×100.

**Figure 5:**
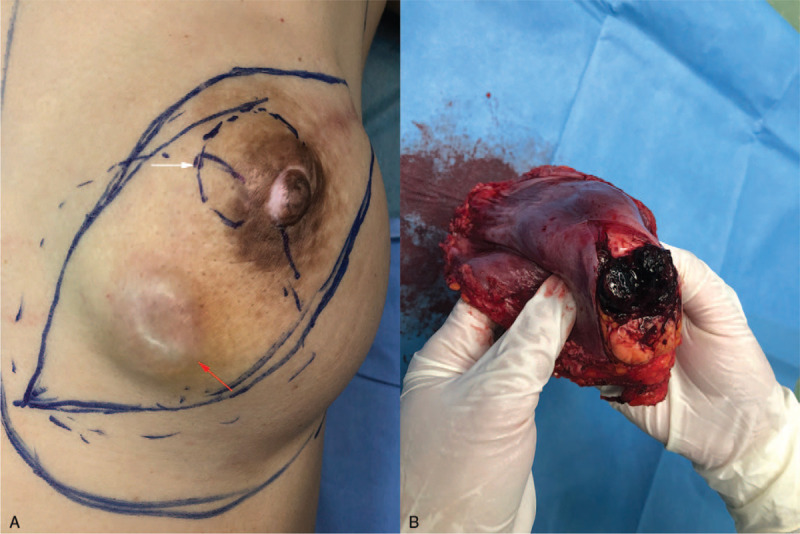
Recurrent angiosarcomas of the left breast. (A) One is located in the lower inner quadrant (red arrow), with apparent bruising of the overlying skin, and a second lesion situated in the left breast (white arrow). (B) Cut off the surface of recurrent tumors.

**Figure 6:**
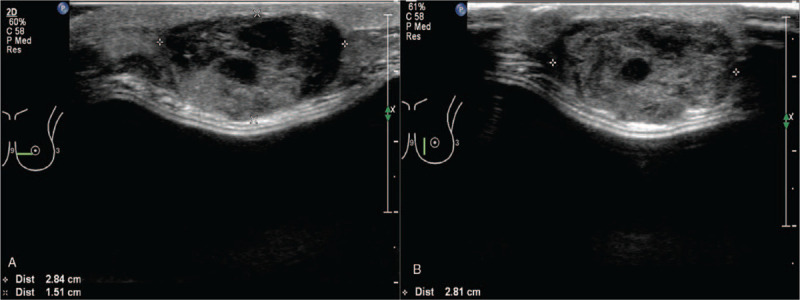
Transverse (A) and longitudinal (B) ultrasonography of the lower inner region of the left breast mass demonstrated a well-defined hypoechoic lesion accompanying anechoic appearance.

**Figure 7:**
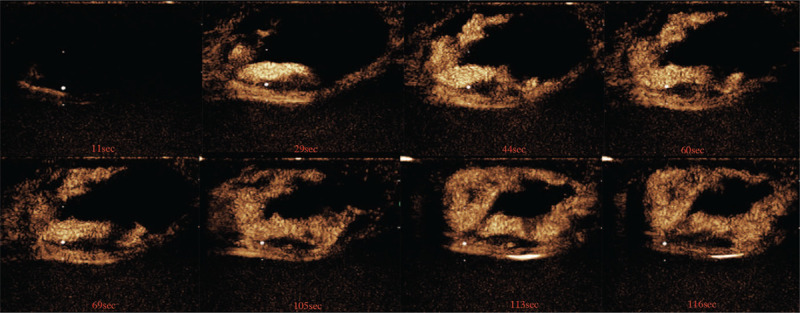
Contrast-enhanced ultrasound images of the left breast lesion after injection of a SonoVue.

The patient underwent a contrast-enhanced breast MRI, and the MRI revealed an irregular mass measuring 6.7 × 6.0 × 4.3 cm in the central and medial region of the left breast, hypointense with scattered mixed high signal intensity foci on T1-weighted images (Fig. 3A), and hyperintense on T2-weighted images (Fig. 3B). The lesion showed a high signal with a high apparent diffusion coefficient value (1.45 × 10 ^−3^ mm^2^/s) in the diffusion-weighted image (Fig. 3C). The time-signal intensity curve (Fig. 3D) revealed gradual and persistent honeycomb-like enhancement (Type-1), which was suspicious of being idiopathic granulomatous mastitis (IGM). An ultrasound-guided core needle biopsy was performed, which was highly suspicious of angiosarcoma. CT scan and bone scan showed no evidence of metastatic disease. The patient received a nipple-sparing simple mastectomy to remove the tumor, and immediate reconstruction of the left breast was performed during the procedure, placing a retro pectoral cohesive gel implant. The mastectomy specimen measured 15.0 × 10.0 × 6.0 cm, and on the section the tumor measuring 7.0 × 7.0 × 5.0 cm, it was soft to firm in consistency, with focal areas of hemorrhage (Fig. 1B). Microscopic examination (Fig. 4A–F) of the mass showed a malignant moderately differentiated vascular neoplasia, consisting of channels lined by endothelial cells. On immunostaining, the tumor cells were positive for CD31, CD34, Ki-67 (+, 40%), focally positive for factor VIII, and negative for E-cad, Calponin, CD10. The final pathology report confirmed breast angiosarcoma grade 2 with free surgical margins and no skin infiltration. The patient refused any additional chemotherapy or external radiotherapy after surgery, and close follow-up was prescribed. The patient noticed a subcutaneous lesion in her left breast's lower inner quadrant 8 months later. The physical examination revealed a 3.0 × 4.0 cm, hard, mobile, and painless mass located in the lower inner quadrant of her left breast (Fig. 5A, red arrow), and with apparent bruising of the overlying skin. A second lesion, 2.0 × 3.0 cm well-circumscribed, firm, and mobile mass, was noted in the periareolar region of the left breast (Fig. 5A, white arrow). Ultrasound of the left breast demonstrated 2 subcutaneous nodules, 1 at the 8 o’clock position (Fig. 6), 5 cm from the nipple measuring 3.0 × 2.8 × 2.5 cm, and a second measuring 2.0 × 1.8 × 1.5 cm at 10 o’clock; both were shown to be well-defined and hypervascular. CEUS was performed for revaluation. On CEUS images, both the 2 masses had an irregular shape and clear margin. It displayed rapid peripheral irregular enhancement with slow washout, an extensive area of central non-enhancement was observed (Fig. 7), and the penetrating surrounding vessels were noted.

**Figure 8:**
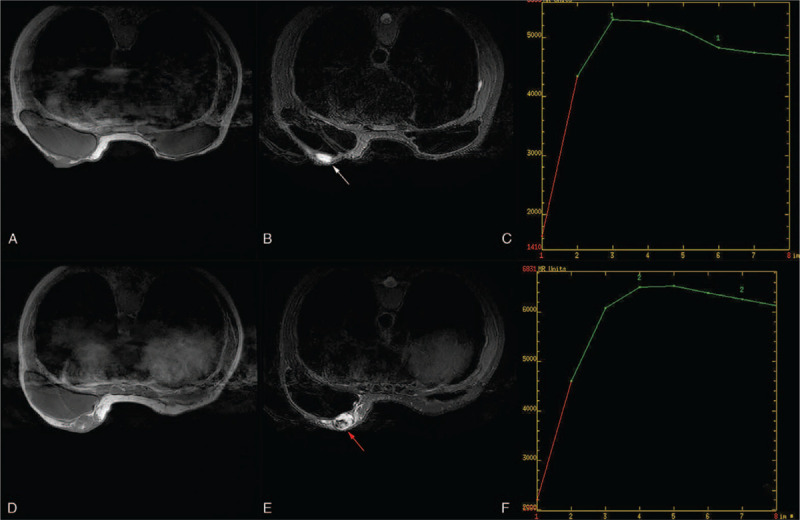
Magnetic resonance imaging of recurrent lesions of the left breast. Both the 2 lesions with (A & D) Low signal intensity on T1-weighted images. (B & E) High signal intensity on T2-weighted images. (C & F) Dynamic contrast-enhanced imaging with time signal intensity curves for type 3.

MRI of the left breast showed the 2 lesions were ill-defined, 1 in the upper inner quadrant, with homogeneous enhancement, and a second lesion in the lower inner quadrant of the left breast, with a significant heterogeneous enhancement of the contrast agent. Both the 2 lesions showed low signal intensity with scattered high signal intensity foci on T1-weighted images (Fig. 8A & D) and high signal intensity on T2-weighted images (Fig. 8B & E). In diffusion-weighted MRI, the lesion showed a high signal with a low apparent diffusion coefficient value (0.85 × 10^−3^ mm^2^/s). The time signal intensity curves (Fig. 8C & F) showed the mass with rapid enhancement and the enhancement curve with a washout pattern (type 3 curve) on contrast MRI. Reexcision of the tumors with implant removal was performed, and on the section of the tumor in the lower inner quadrant of the left breast measuring 3.0 × 3.0 × 2.5 cm, with focal areas of hemorrhage (Fig. 5B), final pathology confirmed the recurrence of angiosarcoma with focal areas of hemorrhage. The patient had a lung metastasis 4 months later. Then she was treated with apatinib, an oral tyrosine kinase inhibitor targeting vascular endothelial growth factor receptor-2 at a daily dose of 250 to 500 mg, showed stable disease for 4 months; she eventually died 22 months after diagnosis.

## 3 Discussion

Angiosarcoma of the breast is a rare and extremely aggressive malignant vascular neoplasm. It may occur as a primary angiosarcoma of the breast or as a secondary lesion induced by chronic lymphedema or radiotherapy after breast conservation.^[6]^ Breast angiosarcoma usually manifests as a palpable, painless mass with a fast-growing rate or painless bruising for large or superficial tumors.^[7]^ Our case appeared with the bruising of the left breast's overlying skin due to a large mass at the first presentation, and the relapsed tumor manifested apparent bruising of the overlying skin because of its superficial location.

Breast angiosarcomas often exhibit no radiographic features on mammograms; it appears most commonly as ill-defined masses, without spiculations or calcifications, and up to one-third of patients show no abnormalities mammograms.^[8]^ Mammography was not planned in our case since the patient declined.

There are no distinctive characteristics specific to angiosarcomas on ultrasound examination, which may mimic benign lesions, leading to the breast's angiosarcoma's diagnostic difficulties. It may present as a hypoechoic, hyperechoic, or heterogeneous mass without acoustic shadowing.^[9,10]^ In our case, ultrasonography revealed a well-defined, isoechoic mass without posterior acoustic shadowing; it was reported as BI-RADS 4A. CEUS is a pure-blood pool imaging technique that can be used in the real-time, continuous observation of the whole process of tumor microcirculation perfusion, which can help diagnosis and differential diagnosis of breast lesions. Most breast cancers enhanced ultrasound mode is characterized by fast forward, heterogeneous enhancement, unclear border, and disordered vessels.^[11]^ Breast cancer lesions with smaller sizes are more inclined to show mild enhancement or moderate enhancement, without blood flow perfusion defect and perforator. While breast lesions with larger sizes often show centripetally high enhancement, irregularly enhanced boundary, inhomogeneous contrast agent distribution up to the peak, the existence of perfusion defects, and perforator vessel.

As for CEUS characteristics of PBA, no cases or series have been described in the English literature. There are only a few pieces of research on CEUS characteristics of primary hepatic angiosarcoma (PHA). The CEUS pattern of PHA manifested peripheral irregular arterial enhancement with remarkable central non-enhancement in some series, and the non-enhancement of the central part of the mass has been attributed to central necrosis, thrombosis, hemorrhage, or fibrosis.^[12,13]^

In our case, the mass was demonstrated as an irregular shape and clear margin on CEUS images. It displayed peripheral irregular rapid enhancement with slow washout, an extensive area of central non-enhancement was observed, the expansion of the enhancement range detected by CEUS range is more significant than the 2-dimensional ultrasound. However, microscopically there were only focal areas of hemorrhage of the tumor in our case, consistent with scattered high signal intensity foci on T1-weighted images. It can not be explained that there was an extensive area of central non-enhancement on CEUS.

Therefore, just like a series of PHA has been described by Wang et al,^[12]^ PBA is rich in variety and irregularly distributed vascular architecture. Most of the internal microvessels are unevenly distributed, the microvessel density around the tumor is more significant than that in the center, the central region of tumors are more likely to be affected due to its distant location, and the blood flow velocity in the central area of tumors may decrease; thus it can present with perfusion defects.^[12]^ MRI usually exhibits hypointense on T1-weighted images and hyperintense on T2-weighted images;^[14,15]^ contrast-enhanced images demonstrate heterogeneously rapid image enhancement and washout.^[16,17]^ High signal intensity on the T1-weighted images may be found, suggesting the presence of slow-flowing blood, or hemorrhage among large masses.^[10]^ Granulomatous mastitis can mimic breast malignancy on clinical, or radiologic examination. Initially, the breast mass was suspicious of being IGM with an MRI examination in our setting. IGM usually demonstrate multiple rim enhancement or clustered ring enhancement patterns when abscesses exist,^[18]^ which did not discriminate well between IGM and honeycomb-like hyperintensity in our case, mostly when time-signal intensity curves were influent.

Both CEUS and MRI of the breast are based on breast nodules’ blood supply and reflect the nodules’ enhanced morphology and hemodynamic characteristics. In our case, the PBA showed irregular peripheral enhancement on both CEUS and MRI, but only central perfusion on MRI. It has been reported that the reason for this difference is that SonoVue can not diffuse into extravascular space, while the MRI contrast agent has a smaller diameter and can penetrate the extravascular tissue.^[12]^ Nevertheless, CEUS showed more inhomogeneous enhancement than MRI. The perfusion defect area of CEUS was more significant than the same section perfusion defect area of MRI. With the addition of CEUS, MRI can differentiate PBA from IGM when high signal intensity on the T1-weighted due to hemorrhage.

Our study has several strengths and limitations. First, it is well documented that PBA can mimic an IGM on MRI examination. Second, in this report, we present the first case of PBA on CEUS in English literature to the best of our knowledge. We described some CEUS features of PBA and compared the enhanced morphology and hemodynamic characteristics of the PBA on CEUS and MRI. The study's weakness is that the present study based on a single institute case limited our ability to summarize PBA's typical sonographic appearance on CEUS.

## 4 Conclusions

It should keep in mind that PBA can simulate as an IGM on MRI images. PBA's diagnostic values with CEUS include peripheral irregular rapid enhancement with slow washout, the presence of an extensive area of central non-enhancement of the mass, and surrounded by radial or perforator vessel. Our study demonstrated that combining a CEUS and MRI to reflect enhanced morphology and hemodynamic characteristics of the PBA could help diagnose just before the operation.

## Author contributions

**Conceptualization:** Hua Luo.

**Data curation:** Zujian Hu, Haibin Xu.

**Formal analysis:** Junling He, Liang Hao.

**Investigation:** Ou Ou Yang, Tian Lan, Junling He, Zujian Hu.

**Methodology:** Tian Lan.

**Project administration:** Haibin Xu.

**Resources:** Chang Shu, Liang Hao.

**Writing – original draft:** Ou Ou Yang, Hua Luo.

**Writing – review & editing:** Hua Luo.

## References

[1] Hui A Henderson M Speakman D Angiosarcoma of the breast: a difficult surgical challenge. Breast 2012;21:584–9. 22305554 10.1016/j.breast.2012.01.001

[2] Costa S Graça SAR Ferreira A Breast angiosarcoma secondary to phyllodes tumour. BMJ Case Rep 2012;2012: doi: 10.1136/bcr-2012-007545. 10.1136/bcr-2012-007545 PMC4544176 23208815

[3] Wang XY Jakowski J Tawfik OW Angiosarcoma of the breast: a clinicopathologic analysis of cases from the last 10 years. Ann Diagn Pathol 2009;13:147–50. 19433291 10.1016/j.anndiagpath.2009.02.001

[4] Yang WT Hennessy BTJ Dryden MJ Mammary angiosarcomas: imaging findings in 24 patients. Radiology 2007;242:725–34. 17325063 10.1148/radiol.2423060163

[5] Chikarmane SA Gombos EC Jagadeesan J MRI findings of radiation-associated angiosarcoma of the breast (RAS). J Magn Reson Imaging 2015;42:763–70. 25504856 10.1002/jmri.24822 PMC4539138

[6] Bennani A Chbani L Lamchahab M Primary angiosarcoma of the breast: a case report. Diagn Pathol 2013;8:66 http://www.diagnosticpathology.org/content/8/1/66 23607567 10.1186/1746-1596-8-66 PMC3651285

[7] Georgiannos SN Sheaff M Angiosarcoma of the breast: a 30 year perspective with an optimistic outlook. Br J Plast Surg 2003;56:129–34. 12791356 10.1016/s0007-1226(03)00025-0

[8] Liberman L Dershaw DD Kaufman RJ Angiosarcoma of the breast. Radiology 1992;183:649–54. 1584913 10.1148/radiology.183.3.1584913

[9] Zemanova M Machalekova K Sandorova M Clinical management of secondary angiosarcoma after breast conservation therapy. Rep Pract Oncol Radiother 2014;19:37–46. 24936318 10.1016/j.rpor.2013.07.013 PMC4056516

[10] Bhosale SJ Kshirsagar AY Patil MV Primary angiosarcoma of breast: a case report. Int J Surg Case Rep 2013;4:362–4. 23466684 10.1016/j.ijscr.2013.01.016 PMC3604700

[11] Li CY Gong HY Ling LJ Diagnostic performance of contrast-enhanced ultrasound and enhanced magnetic resonance for breast nodules. J Biomed Res 2018;32:198–207. 29921747 10.7555/JBR.32.20180015 PMC6103473

[12] Wang L Lv K Chang XY Contrast-enhanced ultrasound study of primary hepatic angiosarcoma: a pitfall of non-enhancement. Eur J Radiol 2012;81:2054–9. 21737220 10.1016/j.ejrad.2011.06.026

[13] Zhao CK Xu HX Guo LH A primary hepatic angiosarcoma mimicking intrahepatic cholangiocarcinoma on conventional ultrasound and contrast-enhanced ultrasound: a case report and review of literatures. Clin Hemorheol Microcirc 2017;66:7–14. 27814287 10.3233/CH-16212

[14] Colwick S Gonzalez A Ngo N Bilateral radiation-induced angiosarcoma of the breast. Breast J 2013;19:547–9. 23865861 10.1111/tbj.12160

[15] Mery CM George S Bertagnolli MM Secondary sarcomas after radiotherapy for breast cancer: sustained risk and poor survival. Cancer 2009;115:4055–63. 19526590 10.1002/cncr.24462

[16] Rosen PP Kimmel M Ernsberger D Mammary angiosarcoma. The prognostic significance of tumor differentiation. Cancer 1988;62:2145–51. 3179927 10.1002/1097-0142(19881115)62:10<2145::aid-cncr2820621014>3.0.co;2-o

[17] Bousquet G Confavreux C Magne N Outcome and prognostic factors in breast sarcoma: a multicenter study from the rare cancer network. Radiother Oncol 2007;85:355–61. 18023492 10.1016/j.radonc.2007.10.015

[18] Yilmaz R Demir AA Kaplan A Magnetic resonance imaging features of idiopathic granulomatous mastitis: is there any contribution of diffusion-weighted imaging in the differential diagnosis? Radiol Med 2016;121:857–66. 27406630 10.1007/s11547-016-0666-x

